# Warm Circumpolar Deep Water transport toward Antarctica driven by local dense water export in canyons

**DOI:** 10.1126/sciadv.aav2516

**Published:** 2020-05-01

**Authors:** A. K. Morrison, A. McC. Hogg, M. H. England, P. Spence

**Affiliations:** 1 Research School of Earth Sciences and ARC Centre of Excellence for Climate Extremes, Australian National University, Canberra, Australia.; 2 Climate Change Research Centre and ARC Centre of Excellence for Climate Extremes, University of New South Wales, Sydney, Australia.

## Abstract

Poleward transport of warm Circumpolar Deep Water (CDW) has been linked to melting of Antarctic ice shelves. However, even the steady-state spatial distribution and mechanisms of CDW transport remain poorly understood. Using a global, eddying ocean model, we explore the relationship between the cross-slope transports of CDW and descending Dense Shelf Water (DSW). We find large spatial variability in CDW heat and volume transport around Antarctica, with substantially enhanced flow where DSW descends in canyons. The CDW and DSW transports are highly spatially correlated within ~20 km and temporally correlated on subdaily time scales. Focusing on the Ross Sea, we show that the relationship is driven by pulses of overflowing DSW lowering sea surface height, leading to net onshore CDW transport. The majority of simulated onshore CDW transport is concentrated in cold-water regions, rather than warm-water regions, with potential implications for ice-ocean interactions and global sea level rise.

## Teaser

Mid-depth heat transport toward Antarctica is focused in “cold” regions, mechanically forced by overflowing dense water.

## INTRODUCTION

Oceanic heat transport across the Antarctic continental slope has a pivotal control on both the global meridional overturning circulation (*1*) and, via glacial ice melt, global sea level (*2*). Warm Circumpolar Deep Water (CDW), which upwells poleward onto the continental shelf, is the primary mechanism that transports heat toward Antarctica’s marine-terminating glaciers. In recent years, warm CDW transport onto the continental shelf has been linked to accelerated basal melting of ice shelves (*3*). Enhanced melting of ice shelves around Antarctica can lead to accelerated glacier flow toward the ocean, as ice shelves provide a buttressing effect on land ice flows (*2**)*. Understanding the local dynamics of the water mass exchanges across the Antarctic continental slope is therefore a globally significant problem.

Observations of ocean transport across the Antarctic continental slope are relatively scarce due to both its remoteness and the frequent presence of sea ice and icebergs. Existing observations are dominated by summer measurements and are focused in particular regions of interest (*4*). Numerical modeling of the circulation around the Antarctic margin is also difficult due to the high resolution required to simulate overflows and a mesoscale field with a typical Rossby radius of ~2 to 5 km along the continental slope (*5*, *6*). As a result, there are few constraints on the processes controlling the cross-slope transports and the dynamical relationship between the transport of different water masses that circulate in this region (*7*).

In response to the recent ocean warming on the Antarctic continental shelf and the accelerated basal melting of Antarctic ice shelves, a number of studies have investigated the mechanisms that drive relatively warm CDW poleward across the shelf break. Here, the term CDW refers to a range of different water masses on the continental slope, including modified CDW, that originate from the middepth warm, salty reservoir in the Southern Ocean. Simple mean geostrophic flows of CDW onto the shelf are not possible due to the sloping bathymetry of the continental slope, away from canyons and other topographic features that could support a zonal pressure gradient. Proposed mechanisms instead include mesoscale eddy transfer along sloping isopycnals (*6*), tidal fluctuations (*8*), interactions between slope currents or topographic waves and canyon features (*9*), bottom Ekman layer transport (*10*), and upward momentum transfer from overflowing dense water (*11*). Because of the difficulty of observing and modeling the Antarctic margin, quantitative estimates of the relative importance of each of these mechanisms are limited (*12*).

Furthermore, the circumpolar spatial distribution of CDW transport onto the shelf is not known even at steady state. While many studies have shown that CDW transport is enhanced within canyons in diverse locations around Antarctica [e.g., (*13*, *14*)], recent work has focused predominantly on CDW transport onto warm-water regions of the shelf. Warm-water regions, such as the Bellingshausen and Amundsen Seas, have a persistent presence of modified CDW on the continental shelf (*7*) and have the most rapidly accelerating ice shelf melt rates (*15*). On the other hand, cold-water regions are characterized by intense atmospheric cooling, high rates of sea ice formation, and, consequently, vigorous dense water production. This dense water production occurs in just a handful of locations around the continent (*16*). A common assumption, for example, as proposed by Schmidtko *et al*. (*3*), is that CDW intrusions are historically more frequent in warm-water regions compared with cold-water regions of the Antarctic continental shelf. However, whether CDW flows onto the shelf relatively uniformly around Antarctica or whether its volume transport is concentrated in particular locations remains unknown.

The other dominant water mass around the Antarctic margin is Dense Shelf Water (DSW), which forms from less-dense waters (primarily upwelled CDW) that are transformed by intense heat loss and brine rejection during sea ice formation near the Antarctic coastline (*16*, *17*). Here, we use the term DSW to cover a range of different dense water masses that arise on the continental shelf and within ice shelf cavities. DSW cascades off the continental shelf in localized overflow plumes [e.g., (*18*)], entraining lighter waters during the descent to form Antarctic Bottom Water, which ventilates the global abyssal ocean and drives the lower cell of the meridional overturning circulation.

Here, we quantify the spatial distribution of cross-slope CDW transport around Antarctica using a global, eddying ocean-sea ice model. We contrast the dynamics of warm-water and cold-water regions of the continental shelf and examine the relationship between DSW export and poleward transport of CDW.

## RESULTS

### Circumpolar overview of water masses and transports

**Fig. 1:**
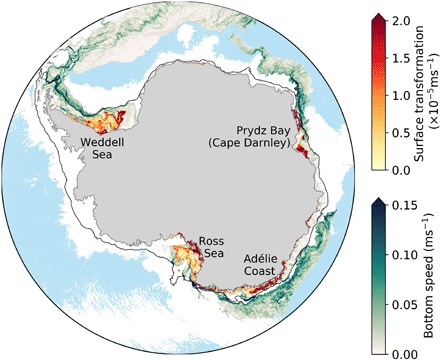
Model simulation of the source locations and descending pathways of dense water. Surface water mass transformation across σ_1_ = 32.57 kg/m^3^ is shown in red colors. Bottom speed is shown in green colors in the descending pathways of dense water (where bottom density σ_4_ >46.105 kg/m^3^). Blue background shading shows bathymetry deeper than 4000 m. The black line represents the 1000-m isobath contour.

The high-resolution (0.1°), global ocean-sea ice model MOM01 (see Materials and Methods) simulates the formation locations and transports of DSW (Fig. 1) very well compared with observations [e.g., (*1*, *16*, *17*)]. A water mass transformation calculation, following Newsom *et al*. (*19*) and using monthly averaged output, gives a maximum surface transformation rate of 6.9 Sv [1 sverdrup (Sv) = 10^6^m^3^s^–1^] over the continental shelf. The formation of dense water is dominated by sea ice formation processes, with 88% of the surface buoyancy flux arising from brine rejection, and only a minor component from surface cooling, consistent with observations (*20*). This is in contrast to most global models, in which Antarctic dense water is commonly formed through open-ocean convection, dominated by surface cooling processes in the center of the Weddell and Ross Seas, rather than through sea ice formation processes over the continental shelf [e.g., (*21*, *22*)]. Models that are able to form dense water over the continental shelf generally have too much mixing in the overflows, resulting in much of the dense water entering intermediate layers rather than descending all the way to the abyss (*21*, *22*). In MOM01, because of the enhanced resolution in both the horizontal and vertical (*23*), shelf-sourced dense water descends all the way to the abyss (Fig. 1), with a bottom water transport of 9.7 Sv at 30°S (as measured by the overturning stream function shown in fig. S1).

**Fig. 2:**
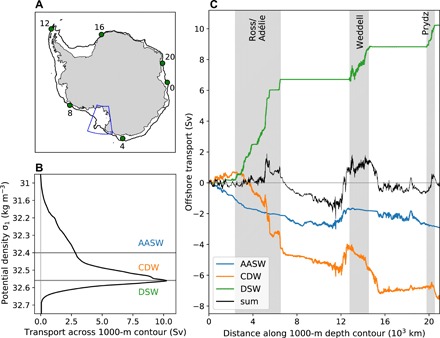
Circumpolar overview of water mass transports across the 1000-m isobath. (**A**) The 1000-m isobath contour, with distances (in 10^3^ km) around Antarctica marked with green circles. The blue box shows the Ross Sea region analyzed in Fig. 3. (**B**) Net offshore transport across the 1000-m isobath, cumulatively integrated upward through density space. The horizontal lines and colored labels show water mass definitions. (**C**) Transport across the 1000-m isobath, cumulatively summed around Antarctica. Colors show transport in different water masses (AASW, Antarctic Surface Water; CDW, Circumpolar Deep Water; DSW, Dense Shelf Water; and the total sum). Distances on the *x* axis are marked on the map in (A). The gray shading in (C) represents the regions where DSW descends the continental slope.

Dense water in MOM01 is formed over the continental shelf in the same four primary locations as identified in observations (*16*): the Ross Sea, along the Adélie Coast, in the Prydz Bay region (known as Cape Darnley in observations), and in the Weddell Sea (Fig. 1). The locations and magnitudes of dense water production are unexpectedly reasonable for a global-scale model, especially given the limitations of the coarse resolution atmospheric forcing that does not resolve katabatic winds. The modeled dense water cascades down the continental shelf, entraining lighter waters, and descending to below 4000-m depth. Figure 2B shows the ocean transport across the 1000-m isobath on the Antarctic continental slope, cumulatively summed upward across density layers. The 10-year average transport is calculated offline using daily averaged output and is conservatively binned into σ_1_ (potential density referenced to 1000 m) layers. The model simulates 10.2 Sv of dense water flowing offshore below a density of σ_1_ = 32.56 kg/m^3^. This transport agrees well with observations, which estimate a net offshore transport of 8.1 ± 2.6 Sv at the upper slope (*24*). The spatial distribution of the offshore DSW transport across the 1000-m isobath is very similar to the spatial distribution of the DSW surface water mass formation (compare the green line in Fig. 2C with Fig. 1); the DSW can thus be seen to flow downslope adjacent to or just downstream from where it is formed on the shelf.

Figure 2C highlights the substantial spatial variability in the simulated CDW transport across the 1000-m isobath (see Materials and Methods for a discussion of water mass definitions, and fig. S2 for a comparison of simulated and observed water mass properties). Some regions have strong poleward flow of CDW (e.g., in the Ross and Adélie sector between ~2 × 10^3^ and 6 × 10^3^ km along the circumpolar contour), while other regions have much weaker flow and do not contribute strongly to the net CDW transport (e.g., the Amundsen and West Antarctic Peninsula sectors between ~7 × 10^3^ and 12 × 10^3^ km along the contour). The regional pattern of strong and weak CDW transport is robust to varying water mass definitions (see Materials and Methods) and is also seen along deeper isobath contours (tested at 1500 m). The circumpolar pattern of heat transport across the 1000-m isobath, calculated relative to the minimum freezing point on the isobath, is qualitatively similar to the pattern of volume transport (fig. S3).

At first glance, it appears counterintuitive that warm-water regions, such as the Amundsen, have only minimal onshore CDW volume and heat transport, compared with the circumpolar integral. For example, the simulated cross-isobath heat transport in the CDW density range between the eastern edge of the Ross Sea and the tip of the Antarctic Peninsula (i.e., a 5500-km span encompassing the Amundsen/Bellingshausen Seas and West Antarctic Peninsula) is only 25 TW. By comparison, over a much smaller 1500-km span across the Ross Sea, the cross-isobath heat transport in the CDW density range is 53 TW. This result is unexpected, because a hypothesis has developed in recent literature that warm-water regions are the dominant contribution to the circumpolar CDW heat transport [e.g., (*3*)].

Comparison of the spatial patterns of CDW and DSW transport in Fig. 2C reveals that the onshore CDW transport is enhanced in the same regions where DSW overflows the shelf. Integrating the CDW transport over just the regions where there is DSW transport (i.e., the three gray shaded regions in Fig. 2C) gives 80% of the net circumpolar CDW transport, despite these regions representing only 32% of the total circumpolar distance around Antarctica. Therefore, while there is large onshore volume and heat transport in the CDW density class in dense water regions, it is largely compensated by offshore DSW volume and heat transport, such that the net cross-isobath volume and heat transport in dense water regions is close to zero. It is possible for the net heat transport to be close to zero in dense water regions, despite high rates of water mass transformation, because the transformation is dominated (88%) by salinity fluxes rather than heat fluxes, and because high rates of mixing over the slope reduce the temperature of CDW as it flows onto the shelf. In warm-water regions (in particular the Amundsen/West Antarctic Peninsula sector), while the onshore CDW transport is considerably smaller, there is no cancellation by overflowing DSW. The resulting depth-integrated net onshore volume and heat transport contributes to the warm temperatures on the shelf in warm-water regions, despite minimal cross-slope CDW transport.

A related process controlling the temperature of warm-water regions is the slow rate of water mass transformation on the shelf. The rate of water mass transformation differs markedly between warm- and cold-water regions, likely influencing residence times and exerting a strong control on the shelf temperature. We hypothesize that the large volume of CDW flowing onto the shelf in cold regions, such as the Ross Sea, is quickly transformed into DSW, while the smaller volume of CDW flowing onto the shelf in warm regions persists for a much longer duration.

The Amundsen/West Antarctic Peninsula region is unique in having a depth-integrated onshore cross-slope volume transport spread along its length (black line in Fig. 2C). This onshore volume transport coincides with the lack of DSW offshore transport in the Amundsen/West Antarctic Peninsula region, combined with the proximity of the eastward flowing Antarctic Circumpolar Current and the absence of a westward Antarctic Slope Current in this region (*7*). It is this sustained net onshore volume transport across the entire Amundsen/West Antarctic Peninsula region that results in the large depth-integrated onshore heat transport in this region (fig. S3). There is one anomalous region, however, near the tip of the Antarctic Peninsula, where there is offshore cross-isobath transport of CDW. This occurs due to the disruption of the westward flowing Antarctic Slope Current at the tip of the Antarctic Peninsula (near location 12 × 10^3^ km along the contour).

In summary, we find that the onshore transport in the Amundsen/West Antarctic Peninsula region is substantial when considering the depth-integrated heat or volume transport (black lines in Fig. 2C and fig. S3C). However, when considering only the CDW density class, the onshore heat and volume transport in warm regions, such as the Amundsen/West Antarctic Peninsula region, is dwarfed by that in dense water regions (orange lines in Fig. 2C and fig. S3C). Given the similarity of the spatial structure of the heat and volume transports, for simplification, we focus on the dynamics of the volume transports only for the remainder of the paper. We now proceed to investigate whether a dynamical link exists between the CDW and DSW transports.

### Regional Ross Sea transport analysis

**Fig. 3:**
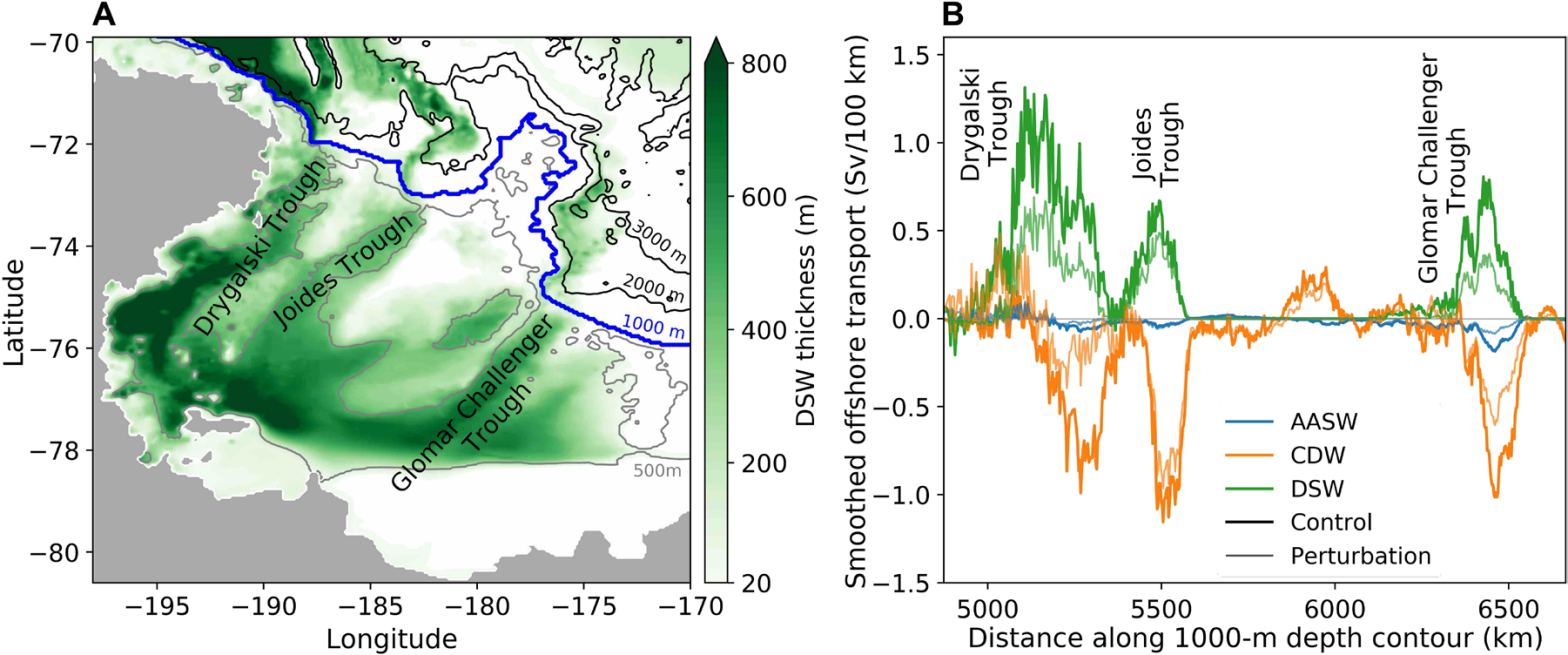
DSW thickness and cross-slope transports in the Ross Sea. (**A**) Average thickness of DSW, showing the location of the overflows aligned with the three canyon features. The blue line shows the 1000-m isobath contour. Black lines show the bathymetry in 1000-m intervals, and gray lines show the 500-m bathymetry contour. (**B**) Downslope transport across the 1000-m isobath in the Ross Sea. A 100-km smoothing filter has been applied to the transports. Dark lines indicate a 10-year average in the control simulation, and pale lines show a 2-year average in the freshwater perturbation simulation.

To investigate the relationship between the CDW and DSW transport in more detail, we zoom in on the 1000-m isobath contour in the Ross Sea (blue box in Fig. 2A), where the model simulates high-dense water production and distinctive overflow features. The dense water overflows the Ross Sea shelf in three locations where there are canyon-like features—Drygalski Trough, Joides Trough, and Glomar Challenger Trough (Fig. 3A). The locations of these three distinct overflows are well captured by the model, and the simulated thickness of DSW on the shelf compares well with observations [see Figure 8a in (*13*)].

The CDW transport onto the Ross Sea shelf is located in the same canyon features as the DSW overflows (Fig. 3B). However, there is a spatial offset between the transports in the two water masses, with the CDW transport located further to the east, with an average offset of ~20 km relative to the DSW. The onshore transport in the Antarctic Surface Water (AASW) layer is small in this region, but is also generally colocated with the overflows, and has a similar eastward offset as the CDW transport. For the remainder of the paper, we focus on the dynamics in the Ross Sea canyons due to the three adjacent overflows with the highest DSW transport in the model. However, similar tight spatial correlations between descending DSW and returning CDW and AASW also occur in other canyons around Antarctica (fig. S4).

**Fig. 4:**
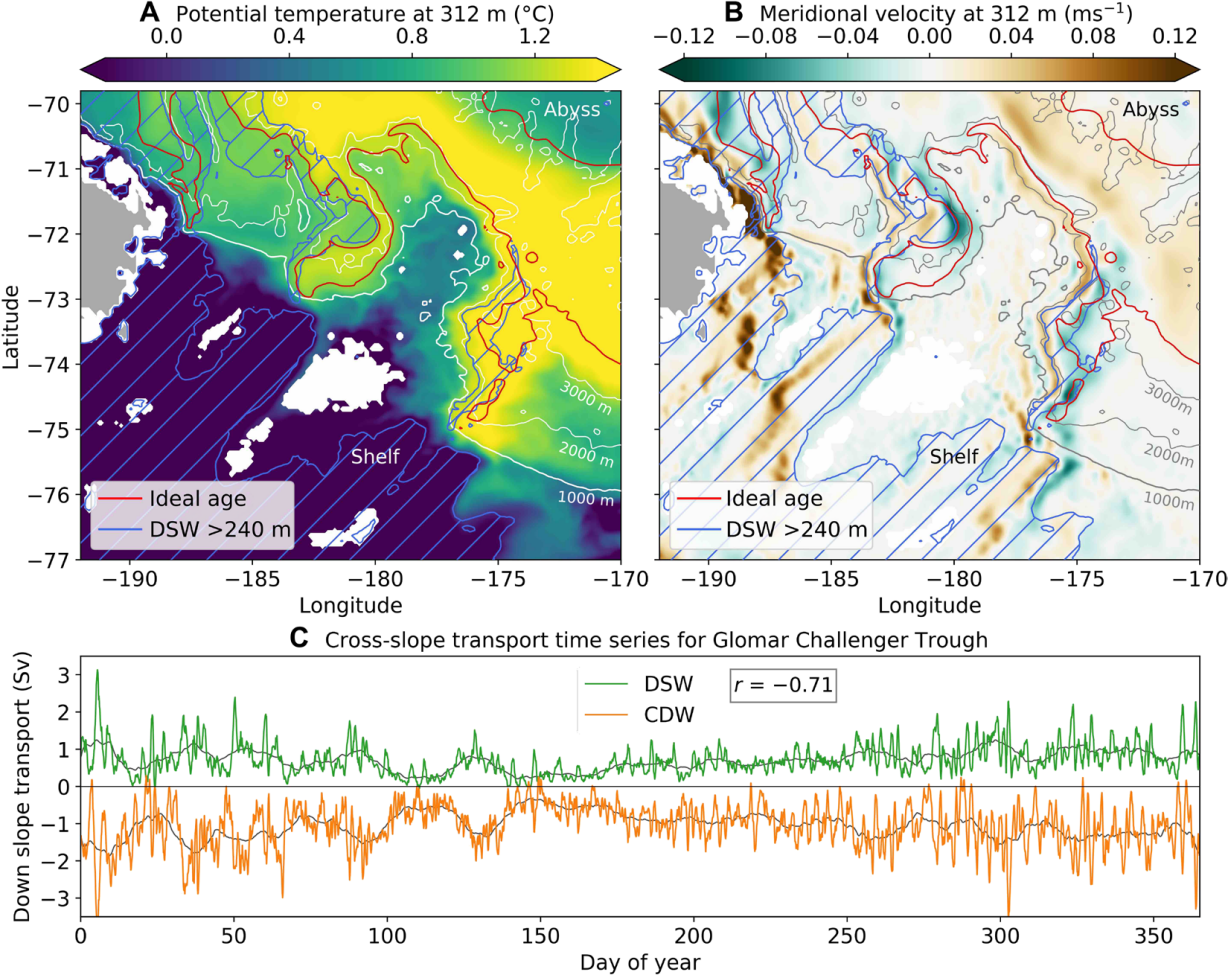
Spatial and temporal correlations between CDW and DSW transport in the Ross Sea. (**A**) Average potential temperature at 312-m depth. (**B**) Average meridional velocity at 312-m depth. Brown (green) shows downslope (upslope) flow. Note that the meridional velocity weakens when the overflow pathway deviates from north/south, as expected. The DSW overflow pathways (DSW thickness >240 m) are shown in (A) and (B) by blue hatched contours. Red contours in (A) and (B) show old waters representative of CDW approaching the shelf (normalized ideal age = 0.85; see Material and Methods for definition). (**C**) A 1-year time series of DSW and CDW transport crossing the 1000-m isobath in the Glomar Challenger Trough (3-hour temporal resolution). The gray lines have been smoothed over a 10-day window to highlight the lower-frequency variability. The correlation coefficient (*r*) is for the raw (3-hour frequency) data, with *P* < 0.01.

The pathways of CDW and DSW across the Ross Sea continental slope are also spatially correlated on a much broader scale beyond the 1000-m isobath (Fig. 4, A and B). In the model, there is a large reservoir of unmodified CDW located offshore beyond the 3000-m isobath (shown by warm, old waters in Fig. 4A). For all three of the canyon features in the Ross Sea, the CDW approaches the shelf edge from the offshore reservoir following the pathway of the DSW overflows (blue hatching in Fig. 4A). As for the transports across the 1000-m isobath, the CDW pathways are offset just to the east of the DSW pathways. The DSW is deflected to the western side of the canyons as it descends, due to the Coriolis force, while the CDW ascends in the center or eastern side of the canyons, depending on the narrowness of the particular canyon. These pathways are sufficiently robust and persistent to be seen in the 10-year average velocity field (Fig. 4B). The velocity within the CDW pathways is toward the shelf in the long-term average, while the water overlying the DSW pathways flows away from the shelf. Similar patterns are seen throughout the water column, suggesting a barotropic nature to these transports.

The simulated overflows consist of episodic pulses of rapidly descending DSW with daily to weekly variability (Fig. 4C). The thickness of dense water in the pulses agrees well with observations of Antarctic overflows. Observations at 1200-m depth in Drygalski Trough, for example, show that a plume thickness of 400 m is common, with values up to 700 m observed (*18*). In the Filchner Depression outflow, dense water pulses were observed to commonly be 100- to 300-m thick over the slope (between 800- and 2500-m depth), with thicknesses up to 800 m (*25*). In the model, the dense water thickness typically reaches a maximum of 300 to 600 m for pulses crossing the 1000-m isobath, depending on the canyon. This is a good agreement between the model and observations, given that we expect discrepancies to exist due to the lack of interannual variability in the model and the fact that the mooring or CTD measurements are unlikely to capture the absolute peak of the overflow pulses because of their limited spatial and temporal resolution. Overflow speeds in the model also compare well with observations. Observations record bottom speeds within plumes of up to 1 ms^−1^ (*18*, *25*). Across the 1000-m isobath in the three Ross Sea canyons, the model simulates typical bottom speeds during overflow events of 0.4 to 0.8 ms^−1^, with occasional large events in the Drygalski Trough reaching >1 ms^−1^. The weaker bottom flow in the model compared with observations is not unexpected due to the relatively coarse vertical grid resolution at the bottom (90 m at 1000-m depth). The repeat frequency of simulated pulses varies between ~2 and 7 days, with individual pulses passing the 1000-m isobath contour in ~1 day. Similar variability has previously been observed in Antarctic overflows (*25*). We have not investigated the mechanisms controlling this variability in detail, but possibilities include topographic vorticity waves (coastal-trapped waves) excited by the descending DSW (*26*, *27*) and baroclinic instability generated at the CDW/DSW interface (*28*). In the ocean, the variability of overflows may also be controlled by tides (*25*).

Figure 4C shows a strong temporal correlation between the transports of CDW and DSW across the 1000-m isobath in the Glomar Challenger Trough. Both the high- and low-frequency variability of the overflows are represented in the CDW time series. The correlation coefficients (*r*) for the three canyons in the Ross Sea are largest for a zero time offset between the CDW and DSW transports and are −0.71 (Glomar Challenger Trough), −0.74 (Joides Trough), and −0.71 (Drygalski Trough), with *P* < 0.01 in all cases. Time series for the other two canyons in the Ross Sea are shown in fig. S5.

The significant spatial and temporal correlations between the CDW and DSW transports hint at a localized dynamical connection, with overflow pulses driving CDW poleward across the continental slope. To test this idea, we performed a separate freshwater hosing perturbation experiment, with the aim of weakening the DSW production and transport, to analyze the response in onshore CDW flow. Additional freshwater was applied during the months of March to October for 5 years. The additional freshwater was added locally in the Ross Sea, roughly targeting the region of high surface water mass transformation (Fig. 1). Because of the response of the sea surface salinity restoring and sea ice to the added freshwater, the net surface freshwater flux also responded to a lesser degree offshore (fig. S6 shows the control and perturbation freshwater fluxes). In the final 2 years of the perturbation, the maximum surface water mass transformation in the Ross Sea shelf region reduced from 2.0 to 0.8 Sv. We analyze the cross-slope transports for the final 2 years of the 5-year perturbation (pale lines in Fig. 3B). While the analysis in Fig. 3B compares simulations of different lengths, due to the atmospheric forcing used, the interannual ocean variability is extremely limited, and a similar result is obtained when the perturbation is compared with the same 2-year period in the control simulation.

The DSW transport in each of the three Ross Sea canyons decreases in response to the reduced dense water formation, along with the onshore CDW transport (Fig. 3B). The canyons with the largest decrease in DSW transport also show the largest decrease in CDW transport. We therefore conclude that the CDW is being dynamically forced onto the shelf by the overflowing DSW.

### Mechanism driving onshore CDW transport

**Fig. 5:**
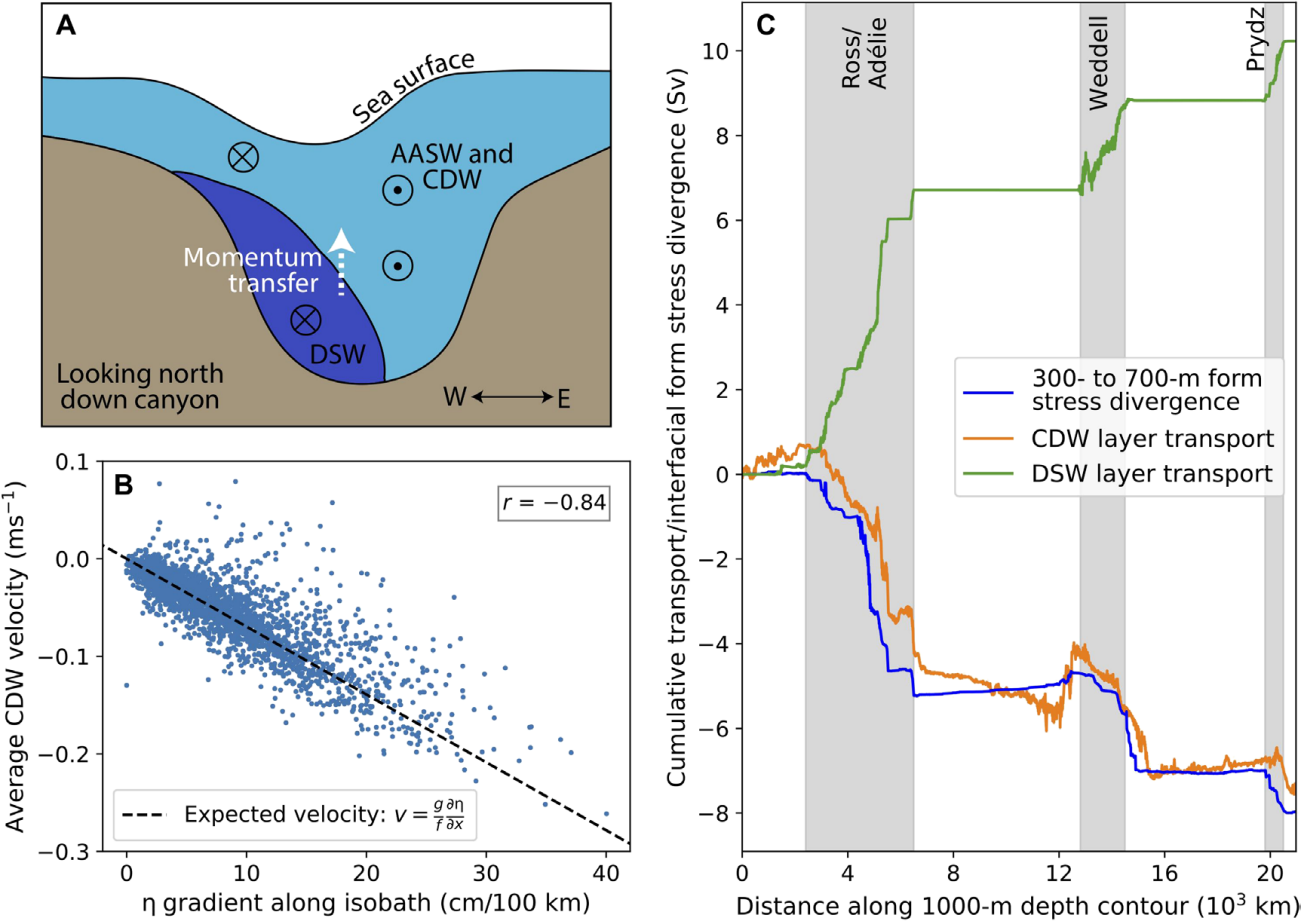
Demonstration of canyon exchange mechanism. (**A**) The schematic view shows a canyon cross section looking toward the open ocean. Dense water hugs the left of the canyon as it descends and lowers the sea surface height above (exaggerated scale). The sea surface variation results in a barotropic pressure gradient that drives AASW and CDW onshore to the east. Directions of flow are indicated by arrow heads and tails. Interfacial form stress transfers momentum upward from the DSW layer to the CDW and AASW layers above. (**B**) Relationship between the 1000-m cross-isobath velocity in the CDW layer and the sea surface height gradient along the 1000-m isobath in the Glomar Challenger Trough. Each point shows a 3-hour average over a 1-year time period. The dashed line shows the expected geostrophic velocity resulting from the barotropic pressure gradient. Downslope velocities are defined as positive. The correlation coefficient (*r*) between ∂η/∂*x* and the simulated model velocity is given. (**C**) The along-isobath cumulative sum of the interfacial form stress divergence summed over middepths (300 to 700 m, blue), compared with cross-isobath water mass transports summed over isopycnal layers.

We propose a simple mechanism to explain the spatial and temporal connection between CDW and DSW transport. As a pulse of DSW descends the continental slope, the dense water displaces less dense water within the canyon. We find that the dominant mode of adjustment is a lowering of the sea surface height above the overflowing pulse of DSW (shown schematically in Fig. 5A). The variation in sea surface height across the canyon results in a barotropic pressure gradient that forces lighter waters (both AASW and CDW) toward the shelf to the east of the DSW and away from the shelf to the west of the DSW. The Coriolis-driven offset of the DSW from the center to the west flank of the canyon results in a net flux of lighter waters toward the shelf. The thickness of CDW and AASW is much greater in the region where the sea surface height slopes upward toward the east in the center of the canyon, compared with the region where the sea surface height slopes upward toward the west on the western side of the canyon (Fig. 5A). Therefore, while there is an offshore transport of CDW in the western part of each canyon, this is overwhelmed by a larger onshore transport of CDW throughout the rest of the canyon. Several of these features are apparent in the analysis that we have presented. The adjacent onshore and offshore flows in the 312-m depth slice (i.e., within the CDW layer) can be seen in Fig. 4B in all three of the canyons in the Ross Sea. The eastward offset of the onshore CDW flow compared with the position of the overflowing DSW is also seen in all three Ross Sea canyons (Fig. 3B) and elsewhere around Antarctica (fig. S4). Last, the barotropic nature of the onshore flow is seen in the similarity between the CDW and AASW transports at each of the canyon features in Fig. 3B and fig. S4. There is a significant spatial correlation between the cross-isobath transport of CDW and AASW in the Ross Sea: *r* = 0.65, *P* < 0.01.

To further test whether the proposed mechanism operates in the model, we now analyze the relationship between the sea surface height (η) gradient and the velocity in the CDW layer. We test the relationship locally, as opposed to averaged across an entire canyon, because the location where the dense water pulses cross the 1000-m isobath is not fixed, and it is also common for multiple dense water pulses to overflow in different locations across the same canyon simultaneously. To test this geostrophic relationship, the temporal anomaly of η from a 3-day average (using 3-hour averaged output) is first calculated in order to eliminate larger scale variations in η due to atmospheric forcing and sea ice melt. This short time scale captures the variations in η associated with the overflows, because the time taken for entire dense water pulses to cross the 1000-m isobath is ~1 day. At each time interval, the largest dense water pulse crossing the 1000-m isobath is located along a 200-km stretch surrounding a particular canyon. The sea surface height gradient along the 1000-m isobath, ∂η/∂*x*, is calculated between the local minimum η, located near the dense water pulse, and the maximum value of η to the east along the isobath, up to 100 km away. The velocity within the CDW layer is averaged over depth and the same lateral span of the 1000-m isobath as ∂η/∂*x*. This analysis is performed on model output with a 3-hour average temporal resolution, over a time span of 1 year.

There is a strong correlation (*r* = 0.84, *P* < 0.01) between the local ∂η/∂*x* and the onshore cross-slope CDW velocity in the Glomar Challenger Trough (Fig. 5B). The simulated CDW velocity reaches magnitudes up to 0.25 ms^−1^ and matches the expected geostrophic velocity resulting from the barotropic pressure gradient. For large overflow events (∂η/∂*x* > 20 cm/100 km), the average η difference between minimum and maximum η values is 6.7 cm over an average span of 28 km. Unfortunately, a comparison with observations is not possible, as presently available observational products of sea surface height in the Antarctic region have a resolution on the order of 80 to 100 km (*29*).

We have only tested the proposed mechanism in the Ross Sea due to the constraints of storing circumpolar, high–temporal resolution data (3-hour averages). Given the strong spatial correlations and similar spatial offset between AASW/CDW and DSW seen in other regions (Fig. 2C and fig. S4), we infer that this mechanism is likely also in play in other overflow regions. However, we note that different canyon geometries (e.g., very wide canyons) or smaller overflow transports in other locations may make detection of the correlations and mechanism more difficult to ascertain, given background variability, such as Antarctic Slope Current instability.

The sea surface height mechanism described is consistent with the form stress mechanism found in the idealized numerical simulations of Stewart and Thompson (*11*). Stewart and Thompson (*11*) found that descending DSW generates substantial form stress at the CDW/DSW interface. The interfacial form stress transfers westward momentum upward into the CDW layer and thereby mechanically forces it southward onto the shelf. The presence of the descending DSW alters the interface heights of the isopycnals above and is reflected at the surface as a depression in the sea surface height. Here, we have chosen to describe the mechanism in terms of the barotropic pressure gradient resulting from the sea surface height variation, rather than through a momentum balance, as this additional framework may be useful for applying to future high-resolution observations of sea surface height. However, we can also test that the divergence of the interfacial form stress is consistent with the cross-isobath CDW transport.

To do this, we diagnose the isopycnal form stress approximately via the cross-isobath eddy buoyancy flux. Assuming geostrophic balance, the interface height displacement, η′, is proportional to the buoyancy perturbation, *b′*, and we can approximate the interfacial form stress (*30*) as

τ≈fv′b′¯(∂b¯∂z) (1)

where *v* is the cross-isobath velocity, and the primes represent deviation from a long-term average. We compute this term using daily output and take variations in *v* and *b* from a 10-year average. Figure 5C shows the isopycnal form stress divergence,−(∂τ/*∂z*)/*f*, summed over the middepth water column (300- to 700-m depth). We do not expect a perfect agreement between the approximate isopycnal form stress divergence and the cross-isobath CDW transport, because the former is summed over the time-averaged depth interval corresponding to the CDW/DSW interface, while the latter is integrated over an isopycnal layer. However, from the close agreement between the two, we infer that the CDW transport may be well explained by the form stress divergence. The middepth form stress divergence is strongly negative in the DSW overflow regions and close to zero elsewhere.

## DISCUSSION

We have found a strong, local dynamical relationship between the export of DSW off the continental shelf and the transport of CDW onto the shelf in an eddy-permitting global ocean-sea ice model. If this mechanism is a true representation of the Antarctic cross-slope exchange in the ocean, it markedly shifts our understanding of where CDW approaches the Antarctic margin.

Recently, efforts to understand Antarctic cross-shelf exchange have focused on climatologically warm regions, such as the West Antarctic Peninsula, due to the large CDW warming and ice-shelf melting trends observed there. However, here, we have shown that there may be only a minimal on-shelf transport of CDW in these warm regions, relative to the circumpolar integrated CDW transport. The presence of warm water on the shelf in these regions may instead be attributed to a minor transport of CDW onto the shelf, combined with weak ocean-atmosphere and ocean-sea ice interactions, likely resulting in a relatively long residence time of CDW on the shelf. On the other hand, in dense water formation regions, such as the Ross and Weddell Seas, we find a large local transport of CDW onto the shelf, but strong water mass transformation leads to a short residence time for the CDW and cold temperatures over the shelf. These large spatial variations in cross-slope transport occur for both heat and volume transport in the CDW density class in the model.

We are unaware of direct observations that can be analyzed to test our modeling results. However, with moorings positioned appropriately in both the east and west side of canyons simultaneously, our proposed mechanism should be relatively straightforward to test. Another indication that the mechanism may be at play in the ocean could be gained through a comparison of a climatological depth slice of a CDW tracer, such as temperature, as shown in Fig. 4A, with the expected or known overflow pathways. While existing climatologies show some hint of CDW approaching the shelf along the pathways of the Glomar Challenger and Joides overflows and spilling onto the shelf in the three Ross Sea canyon locations, the spatial resolution of the observations is insufficient to resolve the pathways in detail [see Figure 6 in (*13*), for example]. This is likely to improve in the coming years with increasing coverage by under-ice Argo and seal data.

While there are no robust observations presently available to test the mechanism, several observational studies report collocated on- and off-shore flows within canyons that are consistent with our study [e.g., (*13*)]. Williams *et al*. (*17*) observed eddy features at the Adélie Sill exchanging CDW with cool shelf waters in the 500- to 1500-m depth range and noted that the eddies appear to be present at times of increased dense water overflow. Using a box inverse model, Snow *et al*. (*31*) found that the onshore flow of CDW across the Adélie Sill is an order of magnitude higher in winter than in summer and suggest that this is driven by the release of available potential energy, as dense water overflows the sill in winter.

The dynamical relationship we have described between the CDW and DSW cross-shelf exchange is also consistent with some previously proposed mechanisms for idealized configurations. For example, Kämpf (*32*) investigated an idealized canyon configuration forced by saline shelf water in both numerical simulations and laboratory experiments. Similar to our results, he found a barotropic up-canyon flow that resulted from the geostrophic adjustment of a density front along the canyon axis. As discussed above, our results are also consistent with the idealized numerical simulations of Stewart and Thompson (*11*), who found that CDW is forced onto the shelf by the vertical momentum flux from descending DSW. Furthermore, the isopycnal connection between the deep ocean offshore and shallow depths on the shelf that occurs in dense water regions allows CDW to access the shelf without any change in potential energy (*33*). These isopycnal connections are also present in our model.

A corollary to our proposed mechanism is that a future reduction in DSW production (e.g., from freshening due to meltwater) may lead to a localized negative feedback on ice shelf melt through reduced CDW heat transport onto the shelf (*34*). However, as illustrated by Silvano *et al*. (*35*), this negative feedback may be limited by a transition from a cold, DSW producing region (with large CDW onshore flow), to a warm region (with weakened CDW onshore flow). The cessation of dense water production would be connected with reduced surface water mass transformation, thereby increasing the residence time of CDW on the shelf, and the region may then shift to a state more similar to the West Antarctic Peninsula with a strong presence of warm waters on the shelf.

Last, we acknowledge the limitations of our model configuration, including the lack of tides and ice shelf cavities, and the resolution that does not fully resolve local mesoscale features. However, the mechanism we describe depends primarily on the presence of episodic dense water overflow pulses and the resultant geostrophic adjustment to these pulses, and not on the nature of how that dense water was formed or what determines its variability. We can, thus, place some confidence in our findings, as the model used here resolves these episodic overflow events and the resultant local geostrophic adjustment. It is possible that other mechanisms that remain unresolved in our model may play an important role in other models or the real ocean, although we would still expect our proposed mechanism to be present due to its fundamental constraint set by both mass continuity and geostrophic adjustment.

## MATERIALS AND METHODS

### Numerical model

The study is based on the analysis of MOM01 [as described in (*23*, *36*)], a global ocean-sea ice model based on the Geophysical Fluid Dynamics Laboratory (GFDL) CM2.6 coupled climate model (*37*). The ocean component of the model is MOM5 (*38*). The atmospheric forcing is derived from version 2 of the Coordinated Ocean-ice Reference Experiments–Normal Year Forcing (CORE-NYF) reanalysis data (*39*). CORE-NYF provides a climatological mean atmospheric state estimate at 6-hour intervals and roughly 2° horizontal resolution, along with representative synoptic variability. Sea surface salinity is restored to a seasonally varying climatology on a 60-day time scale with a piston velocity of 0.16 m day^−1^. The model does not include ice shelf cavities or tides.

MOM01 has 0.1° horizontal resolution and 75 vertical levels. Along the Antarctic continental slope, the model resolution in the zonal direction varies from 2.6 to 5.5 km. The meridional resolution is Mercator north of 65°S but is capped at a minimum of 4.7 km south of this. No mesoscale eddy parameterizations are used, despite the fact that the first baroclinic Rossby radius of deformation in the model varies from 1.8 to 5.5 km along the Antarctic slope. The model’s horizontal resolution is therefore insufficient to completely resolve the mesoscale field in the region of interest, although the model does resolve most of the important bathymetric features (banks and canyons) and meanders in the Antarctic Slope Current.

Unless otherwise specified, the analysis is performed using a 10-year averaging period, following an 80-year spin-up phase. Because of the CORE-NYF forcing, interannual variability is extremely limited in the model simulations. While larger interannual variations may be expected in the real system, the model accurately simulates the high-frequency variability in down-slope DSW transport on local scales (Fig. 4C) that is central to the proposed mechanism.

### Model-observation comparison

We evaluate the simulated water mass structure on the Antarctic continental slope by comparing with observed profiles (fig. S2) from the Marine Mammals Exploring the Oceans Pole to Pole (MEoP) dataset (*40*). The MEoP profiles have maximum instrumental errors of ±0.1°C and ± 0.1 psu (practical salinity unit), and a location accuracy of ~5 km. We select all profiles that lie between the 800- and 1500-m isobaths on the continental slope (profile locations are shown in fig. S2A). In situ temperature is converted to potential temperature. Observed profiles are interpolated onto the model vertical grid and then averaged over the four regions labeled in fig. S2A. No comparison is performed in the Ross Sea sector due to insufficient observations. Model profiles are selected at the same locations as the observed profiles from a monthly climatology of simulated potential temperature and salinity. Portions of model profiles are masked where no observations exist, e.g., below the maximum dive depth of each observed profile, or where any observed variable is missing.

### Water mass definitions

Analyzing the relationships between transports in different layers on a circumpolar scale requires water mass definitions that are applicable in the different ocean states present around Antarctica. Unfortunately, there are no self-evident definitions, because the temperature, salinity, and density of the water masses vary considerably around the Antarctic continent. We therefore strive to keep the definitions simple and widely applicable by using only density thresholds to separate water masses [following (*13*, *17*, *24*, *31*)]. The DSW density cutoff is chosen to capture the maximum possible offshore transport across the 1000-m isobath (e.g., as shown in Fig. 2B). The density threshold separating CDW and AASW is chosen to select the majority of the warmest, oldest waters on the continental slope. This is done using a temperature-salinity plot, as shown in fig. S7 for the circumpolar case. The model’s ideal age tracer, used for color shading in fig. S7 and as red contours in Fig. 4, A and B, obeys the same tracer equation as temperature and salinity, only it is set to 0 at the surface and incremented by the model time step in the interior during the course of the integration. In this way, the age tracer measures the volume-weighted time, since a water parcel was last at the surface. Because of the short duration of the model run, we normalize the age tracer at each output interval by the maximum age in the Southern Ocean, so that a normalized age of 1 corresponds to the oldest waters at that point in the simulation, and 0 corresponds to the youngest waters. The circumpolar definition of CDW captures the large return of onshore flow that occurs within a relatively small density range above the DSW (Fig. 2B). We note that, while we refer to this water mass simply as “CDW,” it also includes modified forms of CDW that are substantially colder and fresher due to mixing as CDW approaches the continental shelf. AASW, flowing seasonally both onshore and offshore, is defined as all waters lighter than CDW.

For regional analyses, we repeat the determination of the water mass definitions using only the slope data from the region of interest. The different density thresholds used for the circumpolar analysis, the Ross Sea analysis, and individual canyon analyses are given in table S1.

We also test the robustness of our analyses to a modified water mass definition for CDW, excluding the coldest waters (potential temperature <−1°C) within the CDW density bounds. These cold waters are instead included in the AASW water mass for this test. Figure S8 (compare with Fig. 2C) shows that the spatial heterogeneity of the CDW transport is qualitatively insensitive to the choice of the AASW/CDW density division, with a similar pattern of strong and weak CDW transport regions around Antarctica for the alternate water mass definition.

## Supplementary Material

aav2516_SM.pdf

## SUPPLEMENTARY MATERIALS

Supplementary material for this article is available at http://advances.sciencemag.org/cgi/content/full/6/18/eaav2516/DC1
