# Supplementary material for: Warm Circumpolar Deep Water transport toward Antarctica driven by local dense water export in canyons
Source: Sci Adv. 2020 May 1;6(18):eaav2516. doi: 10.1126/sciadv.aav2516 (PMC7195130; doi:10.1126/sciadv.aav2516)
Supplement: aav2516_SM.pdf [file aav2516_SM.pdf]

Science Advances
AAAS

advances.sciencemag.org/cgi/content/full/6/18/eaav2516/DC1

Supplementary Materials for

Warm Circumpolar Deep Water transport toward Antarctica driven by local dense water export in canyons

A. K. Morrison*, A. McC. Hogg, M. H. England, P. Spence

*Corresponding author. Email: adele.morrison@anu.edu.au

Published 1 May 2020, *Sci. Adv.* **6**, eaav2516 (2020)
DOI: 10.1126/sciadv.aav2516

This PDF file includes:

Figs. S1 to S8
Table S1

Supplementary Materials

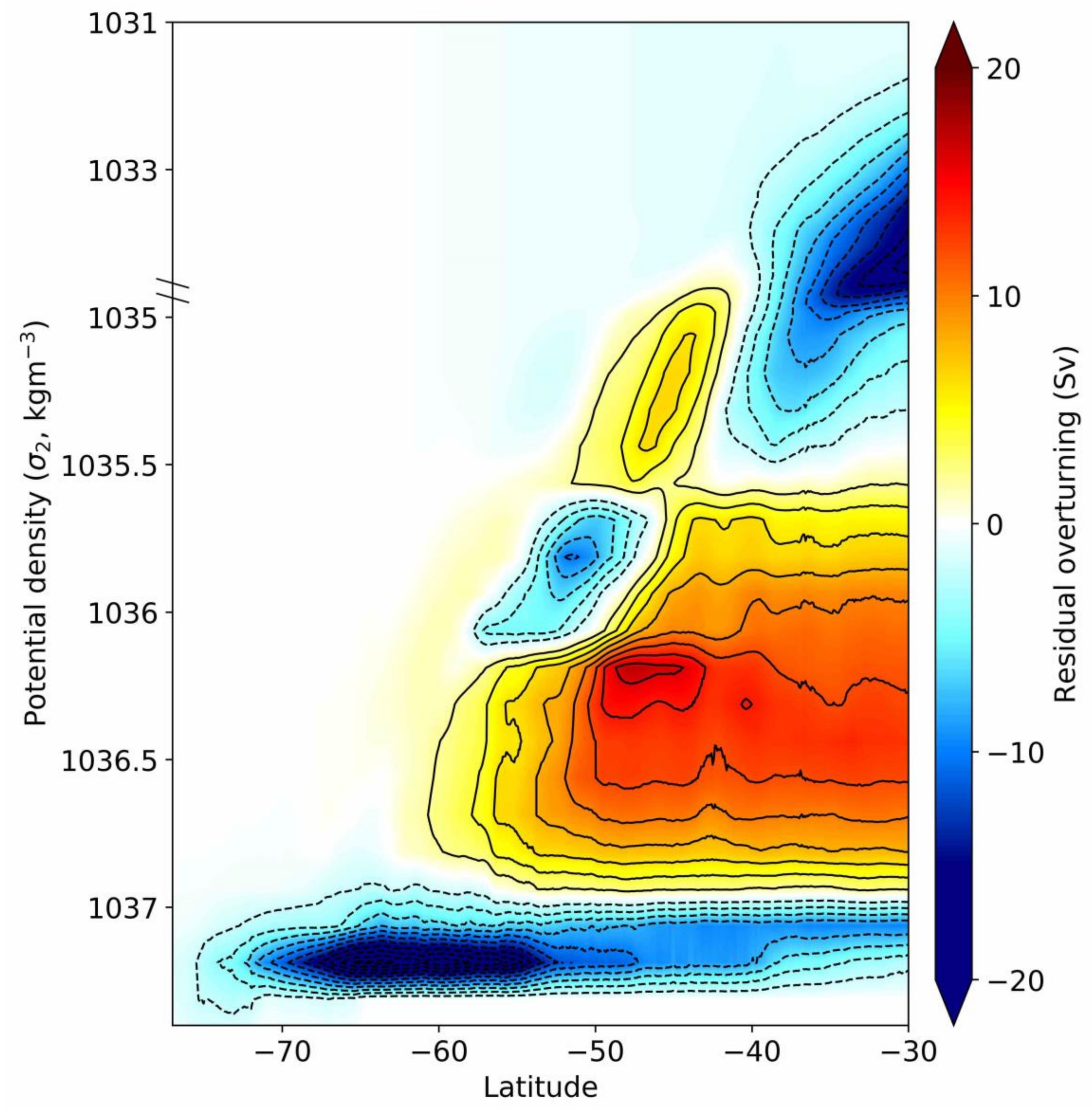

Fig. S1. Southern Ocean overturning streamfunction. Zonally integrated overturning streamfunction in density space, averaged over 10 years. Contours show 2 Sv intervals, and the y-axis is nonlinear.

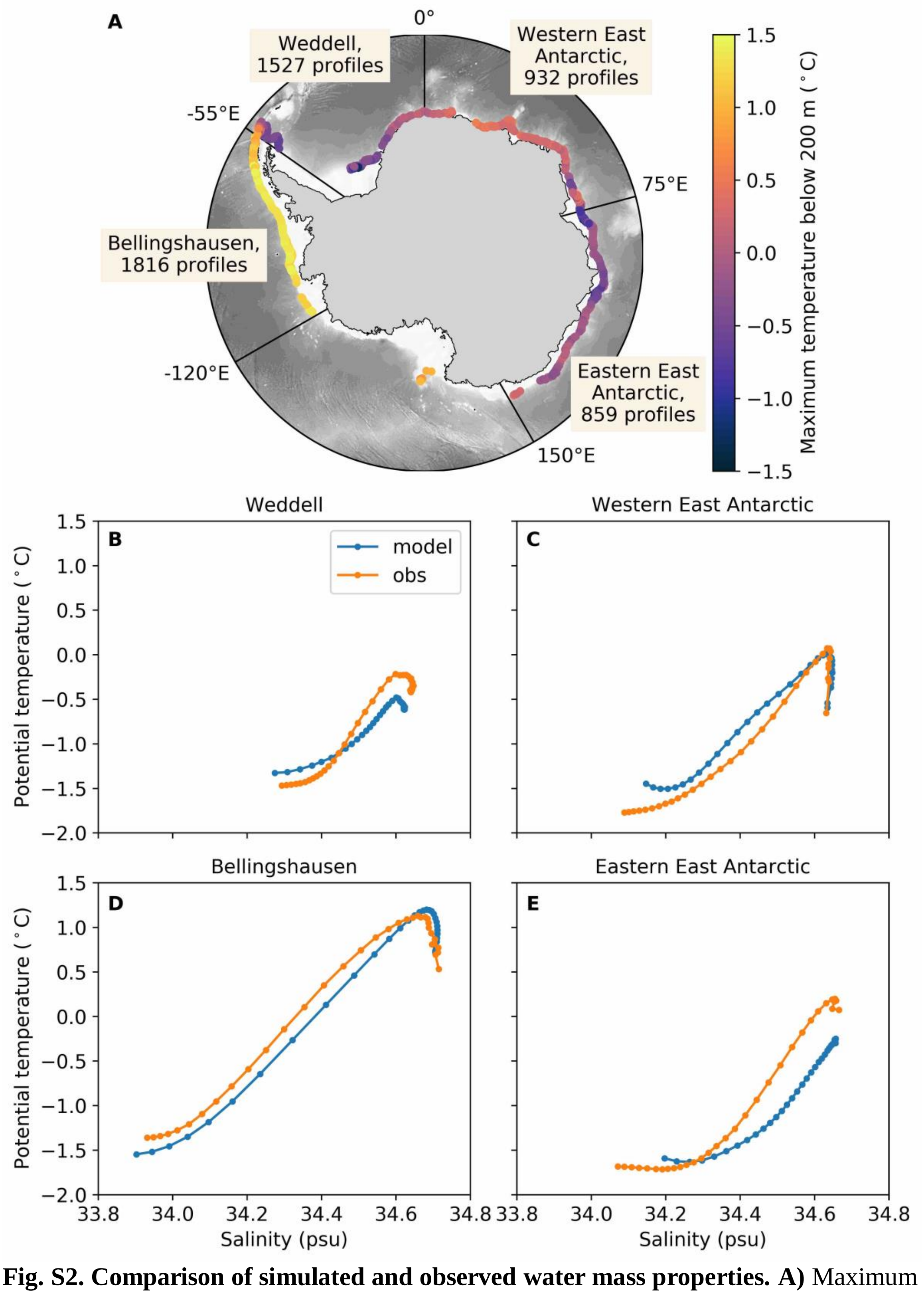

Fig. S2. Comparison of simulated and observed water mass properties. A) Maximum temperature below 200 m in the model at each of the observed profile locations. Region boundaries and labels correspond to panels B-E. **B-E)** Temperature-salinity plots for observed and sub-sampled model profiles below 50 m depth, averaged over the regions labeled in A).

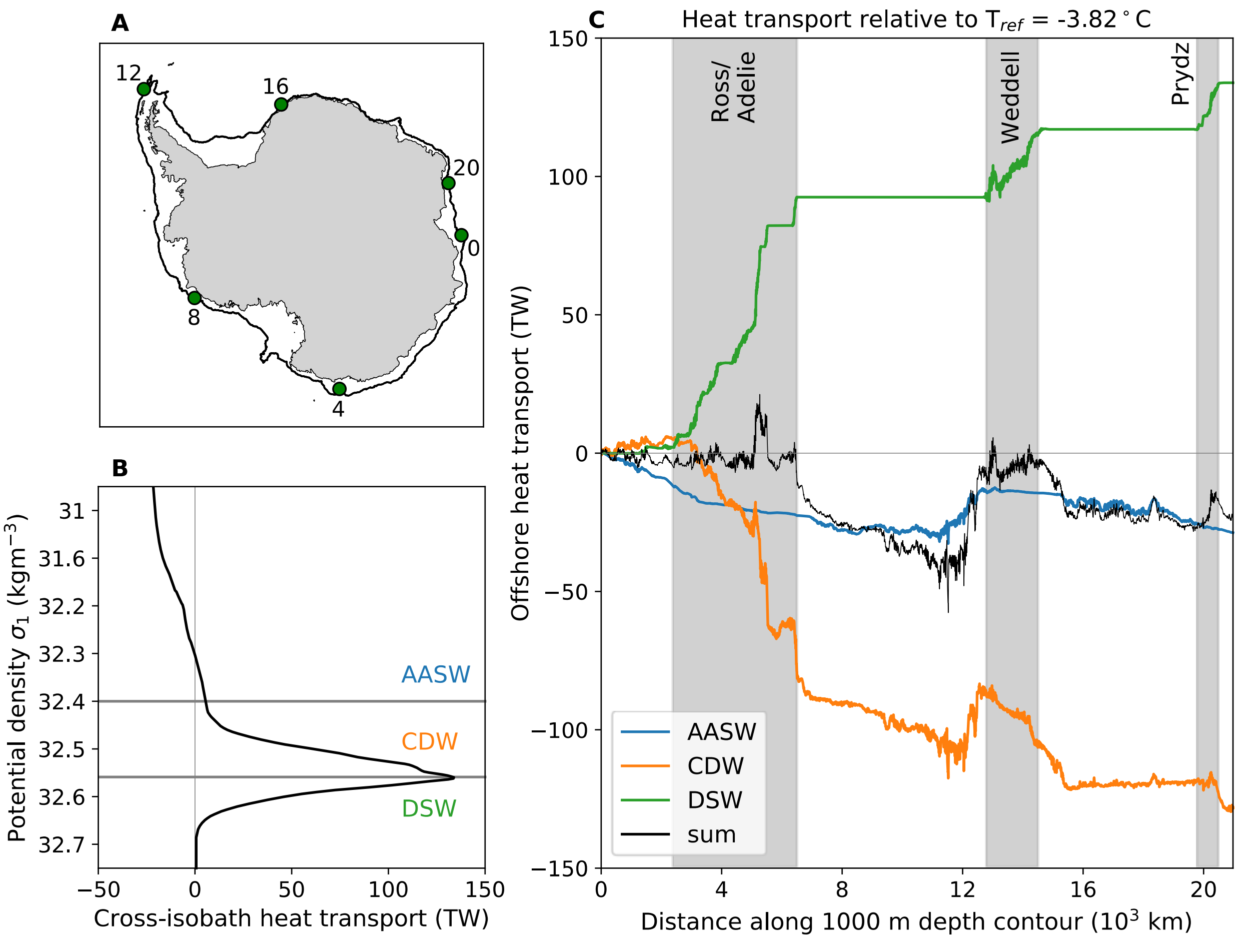

Fig. S3. Circumpolar overview of water mass heat transports across the 1000 m isobath. The heat transports are calculated relative to the minimum freezing point (-3.82°C) on the 1000 m isobath. **A)** The 1000 m isobath contour, with distances (in 10^3 km) around Antarctica marked with green circles. **B)** Net offshore heat transport across the 1000 m isobath, cumulatively integrated upwards through density space. The horizontal lines and colored labels show water mass definitions. **C)** Heat transport, relative to the minimum freezing point, across the 1000 m isobath, cumulatively summed around Antarctica. Colors show heat transport in different water masses (AASW: Antarctic Surface Water, CDW: Circumpolar Deep Water, DSW: Dense Shelf Water, and the total sum). Distances on the x-axis are marked on the map in A). The gray shading in C) represents the regions where DSW descends the continental slope.

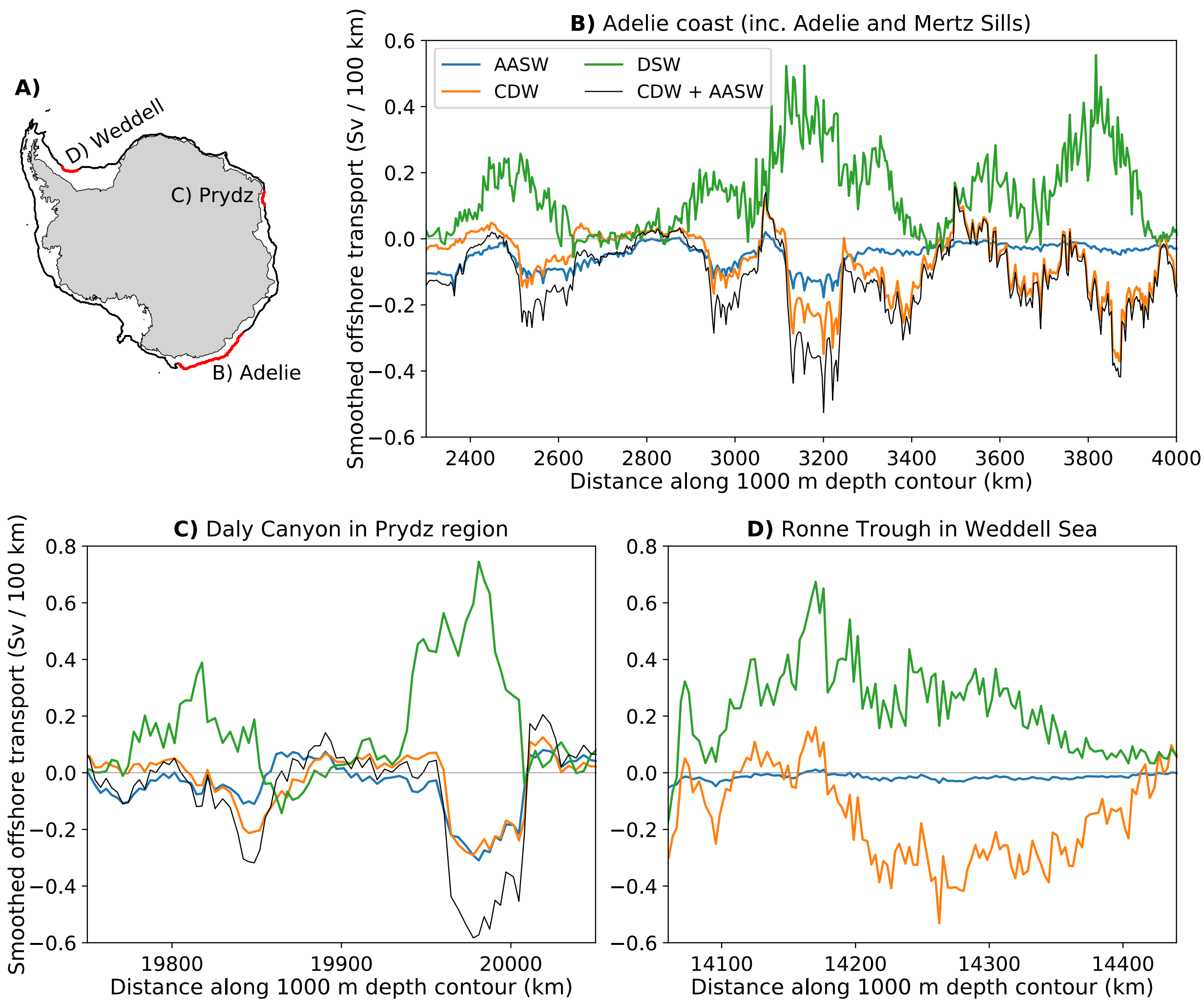

Fig. S4. Cross-slope transports in other dense water regions. A) Map showing the 1000 m isobath contour (black), and the three sections (red) shown in panels B-D. **B-D)** Transport by watermass across the 1000 m isobath along **B)** the Adelie coast, **C)** Daly Canyon in the Prydz region, and **D)** Ronne Trough in the Weddell Sea. These features were chosen because they host some of the largest overflows simulated in the model. A smoothing filter along the isobath has been applied to the transports.

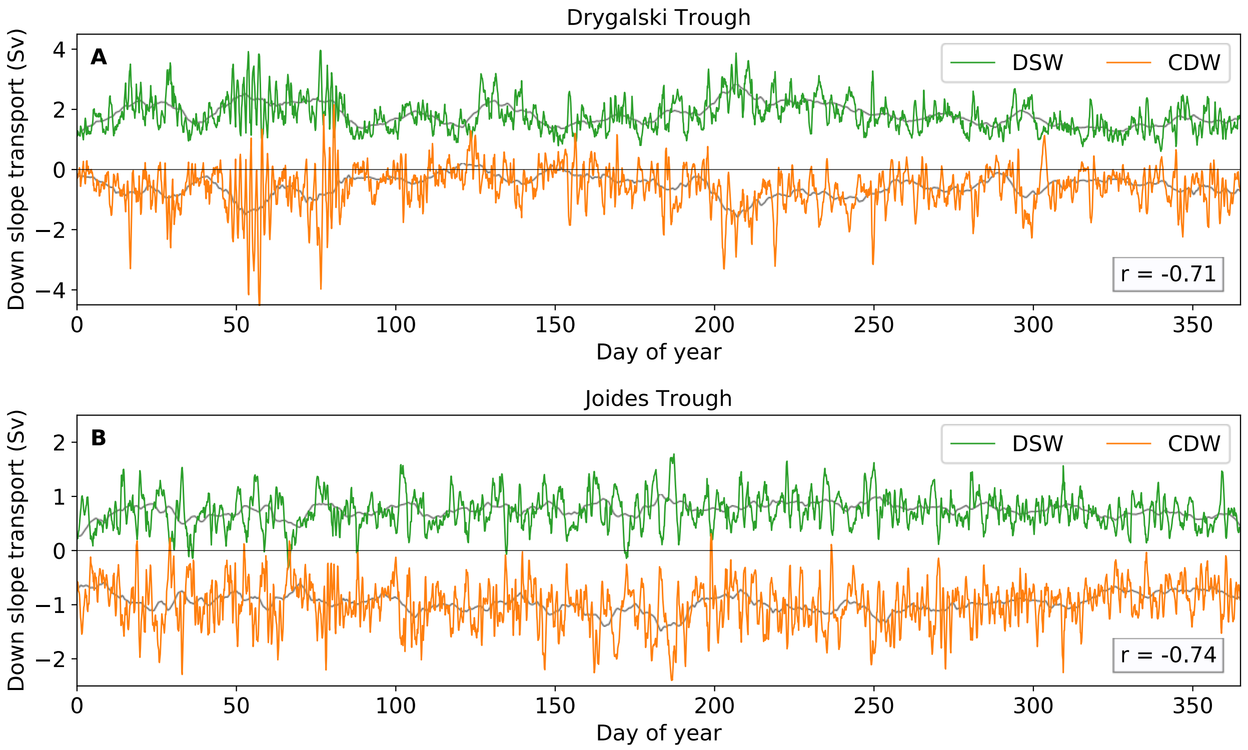

Fig. S5. Time series of cross-slope transports in Drygalski and Joides Troughs. A 1 year time series of DSW and CDW transport crossing the 1000 m isobath in the **A)** Drygalski Trough and **B)** Joides Trough. The model output shown has 3 hour temporal resolution. The grey lines have been smoothed over a 10 day window to highlight the lower frequency variability. The correlation coefficients (*r*) are for the raw (high frequency) data, with $p < 0.01$.

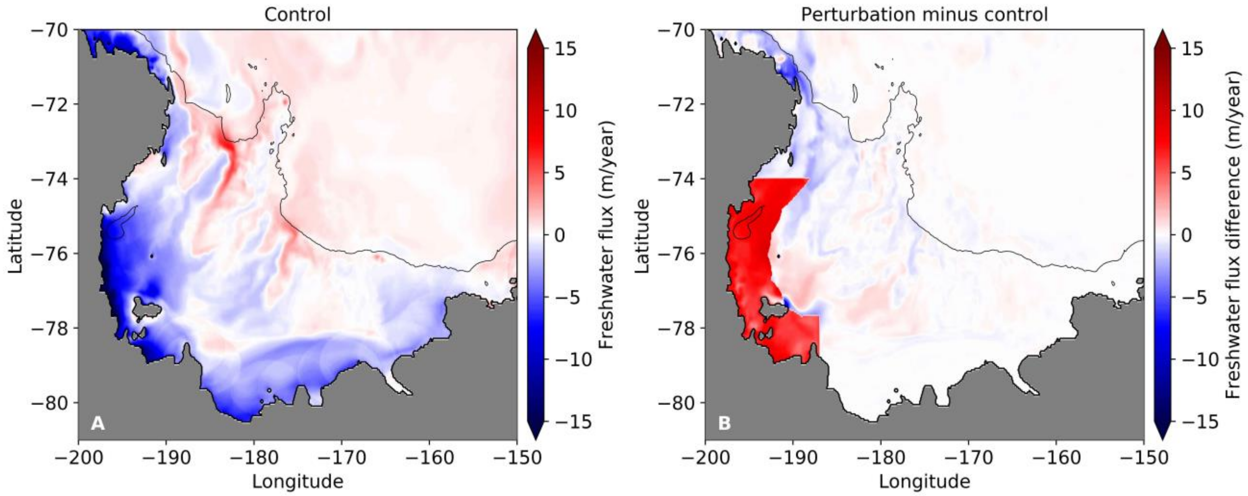

Fig. S6. Ross Sea surface freshwater flux in the control and perturbation simulations. The freshwater flux comprises the sum of atmospheric fluxes (precipitation minus evaporation), sea ice formation and melt, and salinity restoring terms, for **A)** the control simulation and **B)** the freshwater perturbation experiment minus the control. Both panels show a 5 year average over the period of the perturbation.

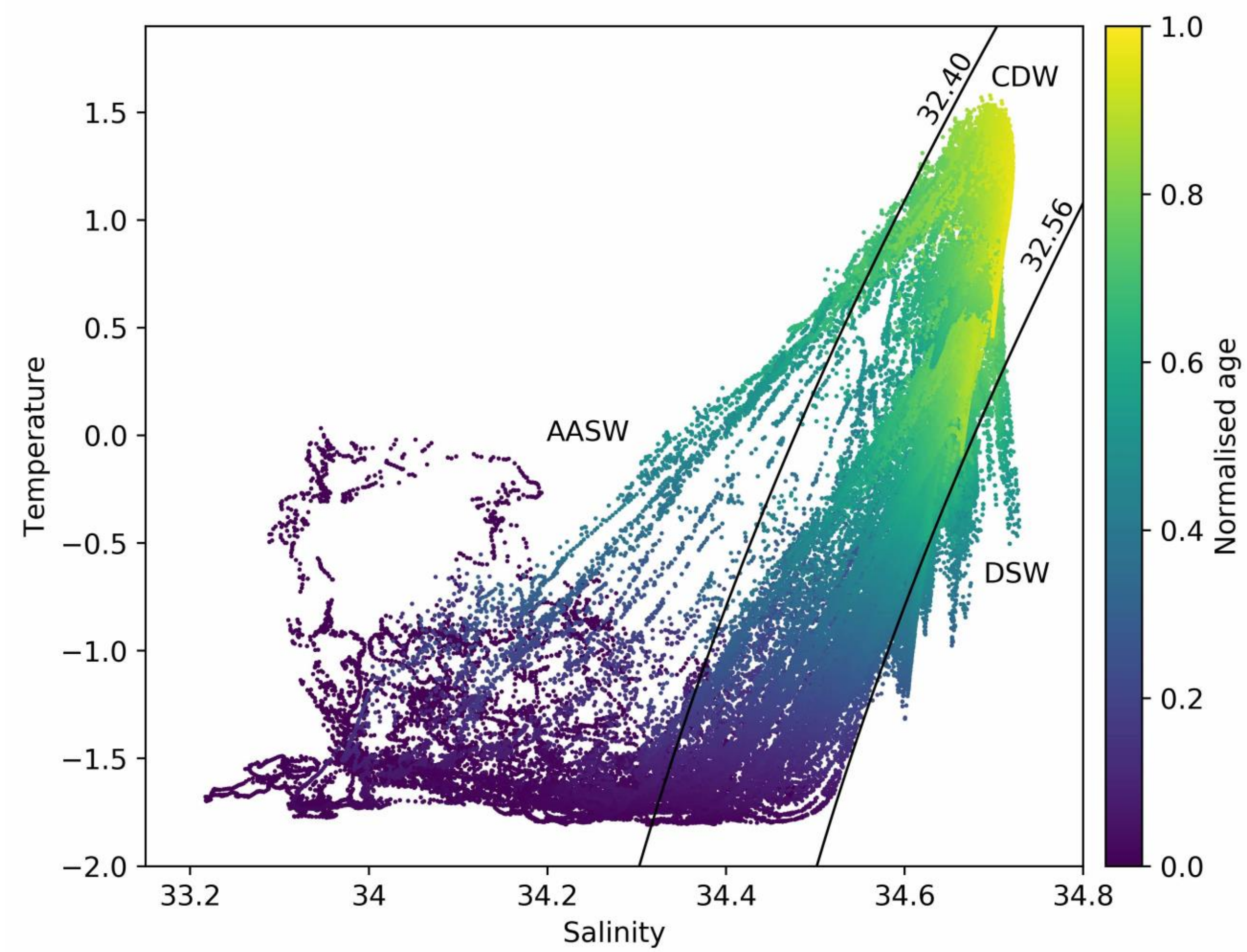

Fig. S7. Circumpolar temperature - salinity plot showing water mass definitions. Each dot represents a 10 year average of a 50 m thick layer at a single latitude/longitude model grid point along the 1000 m isobath contour. Colors indicate ideal age tracer, which has been normalized by the maximum age in the Southern Ocean, so that yellow corresponds to the oldest waters and blue to the youngest waters. Note that the fresh end of the salinity axis is nonlinear.

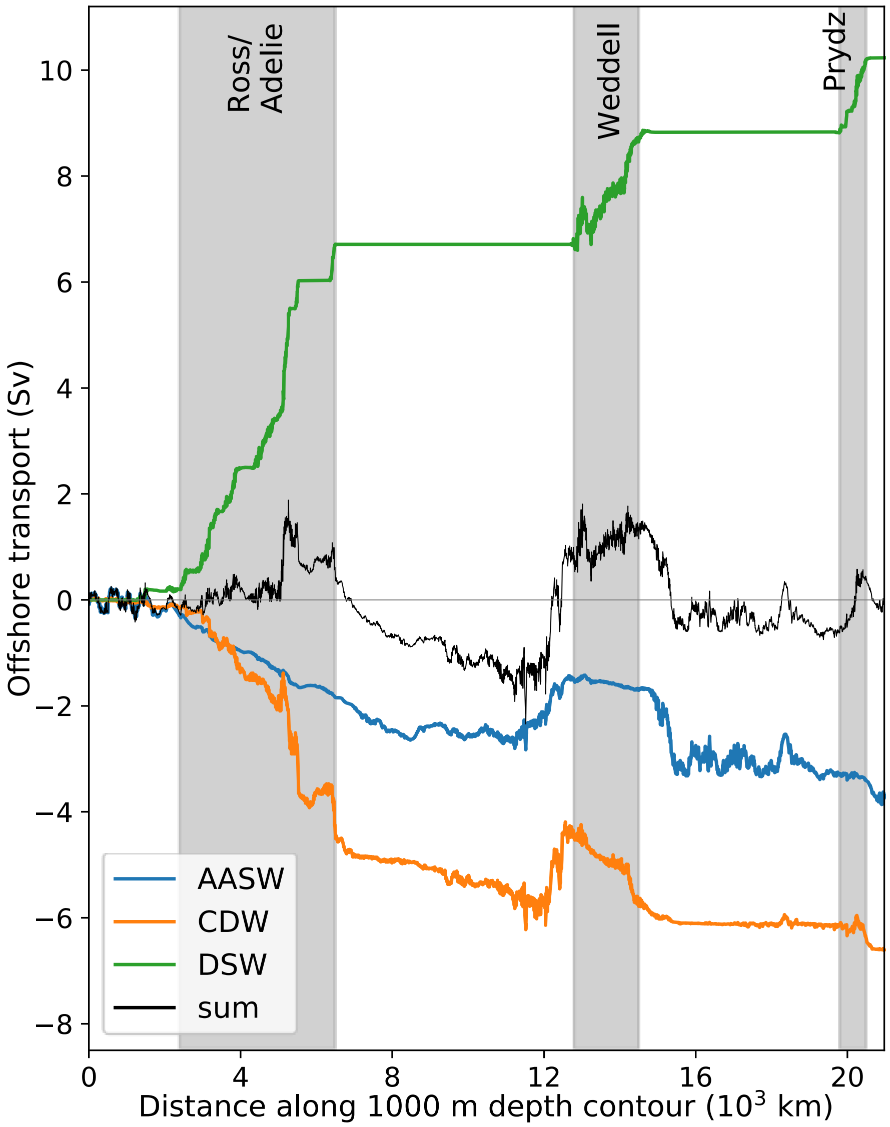

Fig. S8. Circumpolar cross-slope transport using alternative water mass definitions. The same analysis as shown in Fig. 2C (cumulative sum of transport along the 1000 m isobath contour), except with an additional water mass criterion for CDW: only waters with temperature > -1°C are included within the density bounds for CDW shown in Fig. S5. Waters within the CDW density range, but with temperatures < -1°C are included instead in the AASW water mass. Transport using these alternate water-mass definitions is calculated across the 1000 m isobath, cumulatively summed around Antarctica. Colors show transport in the different water masses. The gray shading represents the regions where DSW descends the continental slope.

Region	DSW/CDW<br>σ_1 separation (kgm^{-3})	AASW/CDW<br>σ_1 separation (kgm^{-3})
Circumpolar (Figs. 2, S5)	32.56	32.40
Ross Sea (Fig. 3)	32.53	32.43
Glomar Challenger Trough (Figs. 4C, 5B)	32.57	32.42
Drygalski Trough (Fig. S3A)	32.59	32.42
Joides Trough (Fig. S3B)	32.53	32.44

Table S1. **Density thresholds used for water mass definitions.**
